# YAP/TAZ regulates TGF-β/Smad3 signaling by induction of Smad7 via AP-1 in human skin dermal fibroblasts

**DOI:** 10.1186/s12964-018-0232-3

**Published:** 2018-04-25

**Authors:** Zhaoping Qin, Wei Xia, Gary J. Fisher, John J. Voorhees, Taihao Quan

**Affiliations:** Department of Dermatology, University of Michigan Medical School, 1301 Catherine, Medical Science I, Room 6447, Ann Arbor, MI 48109-0609 USA

**Keywords:** YAP, TAZ, TGF-β/Smad, Smad7

## Abstract

**Background:**

Transcription factors YAP and TAZ function as the primary mediators of the Hippo pathway. Yet, crosstalk of YAP and TAZ with other signaling pathways remains relatively unexplored. We have explored the impact of YAP and TAZ levels on the TGF-β/Smad signaling pathway in human skin dermal fibroblasts.

**Methods:**

YAP and TAZ levels in dermal fibroblasts were reduced in dermal fibroblasts by siRNA-mediated knockdown. The effects of YAP and TAZ reduction on TGF-β/Smad signaling were examined by quantitative real-time PCR, Western analysis, and immunostaining. Luciferase reporter assays and electrophoretic mobility shift assays were conducted to investigate the transcription factor DNA-binding and transcriptional activities.

**Results:**

Knockdown of both YAP and TAZ (YAP/TAZ), but not either separately, impaired TGF-β1-induced Smad3 phosphorylation and Smad3 transcriptional activity, thereby inhibiting the expression of TGF-β target genes. This reduction by reduced levels of YAP/TAZ results from induction of inhibitory Smad7, which inhibits Smad3 phosphorylation and activity by TGF-β1. Conversely, prevention of Smad7 induction restores Smad3 phosphorylation and Smad3 transcriptional activity in fibroblasts that have reduced YAP/TAZ. In agreement with these findings, inhibition of YAP/TAZ transcriptional activity, similar to the reduction of YAP/TAZ levels, also significantly induced Smad7 and impaired TGF-β/Smad signaling. Further investigations revealed that reduced levels of YAP/TAZ led to induction of activator protein-1 (AP-1) activity, Activated AP-1 bound to DNA sequences in the Smad7 gene promoter, and deletion of these AP-1 binding sequences substantially reduced Smad7 promoter reporter activity.

**Conclusion:**

YAP/TAZ functions in concert with transcription factor AP-1 and Smad7 to regulate TGF-β signaling, in human dermal fibroblasts. Reduction of YAP/TAZ levels leads to activation of AP-1 activity, which induces Smad7. Smad7 suppresses the TGF-β pathway.

**Electronic supplementary material:**

The online version of this article (10.1186/s12964-018-0232-3) contains supplementary material, which is available to authorized users.

## Background

YAP and TAZ are central downstream effectors of the Hippo signaling pathway, which plays a crucial role in growth control, proliferation and tumor suppression [1, 2]. YAP and TAZ share substantial sequence homology and often exert similar, overlapping functions. For these reasons, they are often referred to together as YAP/TAZ. YAP/TAZ activity is primarily regulated by sub-cellular localization, which is responsive to cell-cell interactions through the hippo pathway [3] or mechanical forces through unknown mechanisms [4]. Retention of YAP/TAZ in the cytoplasm, due to phosphorylation and/or binding to other proteins inhibits nuclear translocation and transcriptional co-activator function. Many aspects of YAP/TAZ including its role in key cellular processes, regulation, and mechanism of action as a transcriptional co-activator are the subjects of intensive study. However, functional cross-talk of YAP/TAZ with other signaling pathways and transcription factors remain relatively unexplored.

TGF-β is a multifunctional cytokine that regulates numerous developmental and homeostatic processes [5, 6]. TGF-βs initiate their cellular functions by binding to the cell surface TGF-β receptor complex, which triggers activation of intracellular signaling molecules known as Smads. There are three classes of Smad proteins: regulatory (R)-Smads, co-activator (Co)-Smads, and inhibitory (I)-Smads. R-Smads (Smad2 and Smad3) are phosphorylated by the TGF-β type I receptor and form heteromeric complexes with the Co-Smad, Smad4, which translocate to the nucleus and bind to Smad-binding elements (SBE) to regulate transcription of target genes. Phosphorylation of Smad2/3 by TGF-β receptor complexes is antagonized by the endogenous inhibitory Smad, Smad7 [7, 8]. Expression of Smad7 is inducible by TGF-β itself, and this induction plays a critical role in a negative feedback mechanism that regulates TGF-β/Smad biological responses. Smad2 and Smad3 are the primary mediators of TGF-β signaling. Aberrant expression of Smad7 could upset the delicate balance inherent in TGF-β/Smad signaling and thereby contribute to the pathophysiology of certain human diseases. For example, in the fibrotic disease scleroderma, the level of Smad7 expression has been reported to be reduced [9]. This reduction could permit unrestrained activation of the TGF-β/Smad pathway, resulting in excess production and deposition of collagen. Conversely, Smad7 has been reported to be over-expressed in inflammatory bowel disease [10]. This over-expression could down-regulate TGF-β-mediated immunosuppression, resulting in hyper-secretion of proinflammatory cytokines.

TGF-β/Smad pathway is also recognized as a primary regulator of extracellular matrix (ECM) homeostasis [11, 12]. Deregulation of TGF-β signaling plays a prominent role in the pathogenesis associated with ECM proteins. For example, up-regulation of TGF-β signaling causes abnormal accumulation of ECM proteins in affected tissues, as seen in systemic sclerosis (SSC), a chronic and progressive connective tissue disease characterized by fibrosis of the skin and internal organs [13, 14]. In contrast, TGF-β signaling is often perturbed in aged human skin due to reduced expression of TGF-β type II receptor (TβRII) and Smad3 activation [12, 15]. Impaired TGF-β signaling negatively regulates collagen homeostasis and has a significant impact on human skin connective tissue aging [12, 16]. As such, aberrant TGF-β signaling is closely linked to the pathophysiology of broad human diseases. Here we report that YAP/TAZ modulates TGF-β/Smad3 pathway through a distinct mechanism in human skin primary fibroblasts. Low levels of YAP/TAZ or inhibition of YAP/TAZ nuclear translocation lead to up-regulation of Smad7 expression via AP-1 transcription factor. Elevated Smad7, in turn, inhibits Smad3 phosphorylation and impairs TGF-β/Smad pathway. These data support the concept that YAP/TAZ functions as an endogenous repressor of Smad7 expression to modulate TGF-β signaling.

## Methods

### Materials

**Fig. 1:**
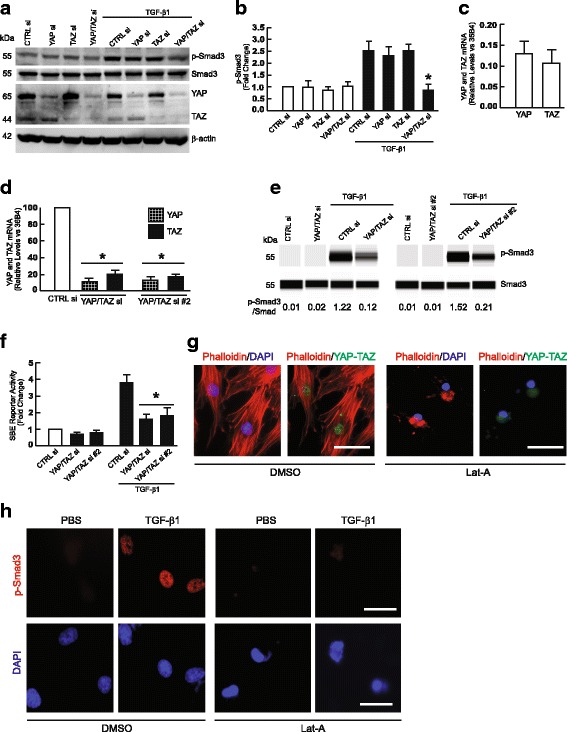
YAP/TAZ knockdown impairs TGF-β1-induced Smad3 phosphorylation and Smad Binding Element (SBE) luciferase reporter activity. Primary adult human dermal fibroblasts were transfected with non-specific control siRNA or YAP and/or TAZ siRNAs (400 nM). 48 h after transfection, cells were treated with vehicle (PBS) or TGF-β1 (5 ng/ml) for one hour and then whole cell extracts were prepared for analyses. **a** Protein levels of phospho-Smad3, total Smad3, YAP, and TAZ were determined by Western blots. Images represent four independent experiments. Full-length blots are presented in Additional file 1: Figure S2. **b** Quantification of Phospho-Smad3 protein levels. Protein levels were normalized by total Smad3. *N* = 4, *p < 0.05 vs TGF-β1 control. **c** Relative mRNA levels of YAP and TAZ in adult human primary dermal fibroblasts. *N* = 5. **d** Knockdown levels of YAP and TAZ mRNA by two different sets of YAP and TAZ siRNAs. *N* = 3, *p < 0.05 vs CTRL siRNA. **e** Protein levels of total and phospho-Smad3 were determined by capillary electrophoresis immunoassay and normalized to total Smad3. Band intensities were quantified by Compass software. Bands show representative digital images. **f** YAP/TAZ knockdown inhibits TGF-β1-induced SBE luciferase reporter activity. Fibroblasts were co-transfected with non-specific control or YAP/TAZ siRNAs (400 nM) and Smad Binding Element (SBE) luciferase reporter, and β-galactosidase expression vector. 32 h later, cells were treated with vehicle (PBS) or TGF-β1 (5 ng/ml) for 16 h. SBE luciferase activities were determined 48 h after transfection and normalized to β-galactosidase activity. Bars indicate fold change in SBE reporter activity relative to control siRNA. *N* = 3, *p < 0.05 vs control siRNA with TGF-β1. **g** Actin cytoskeleton disassembly inhibits YAP/TAZ nuclear localization. Fibroblasts were treated with vehicle (DMSO) or latrunculin A (Lat-A, 30 nM) for 24 h and then F-actin was stained with phalloidin (red), YAP/TAZ were immunostained (green), and nuclei were stained with DAPI (blue). Scale bars = 25 μm. All images are representative of three independent experiments. **h** Actin cytoskeleton disassembly inhibits TGF-β1-induced Smad3 phosphorylation. Fibroblasts were treated with DMSO or Lat-A (30 nM) for 24 h, and then cells were treated with PBS or TGF-β1 (5 ng/ml) for one hour. Smad3 phosphorylation (p-Smad3) was determined by immunostaining (red). Nuclei were stained with DAPI (blue). Scale bars = 25 μm. All images are representative of three independent experiments. All data are mean±SEM

Dulbecco’s Modified Eagle’s Media (DMEM), αMEM, GlutaMAX™, fetal calf sera, trypsin solution, penicillin/streptomycin, and L-glutamine were purchased from Life Technology (Rockville, MD). [γ-^32^p]ATP and [α-^32^p]dCTP were obtained from New England Nuclear Life Science Products (Boston, MA). Smad3, Smad7, CCN1/CTGF, YAP, TAZ, and MMP-1 antibodies were purchased from Santa Cruz Biotechnology (Santa Cruz, CA). Phospho Smad3 antibody for Fig. 1a was purchased from Cell Signaling Technology (Danvers, MA). Phospho Smad3 antibody for Fig. 3d was purchased from Abcam (Cambridge, MA). β-actin antibody was purchased from Sigma Chemical Company (St. Louis, MO). RNA extraction kits were from Qiagen (Chatsworth, CA). Reagents for real-time PCR were from Applied Biosystems (Foster City, CA). Protein Assay Kit was from Bio-Rad Laboratories (Hercules, CA). All other reagents were purchased from Sigma Chemical Company (St. Louis, MO).

### Cell culture and ethics statement

Human skin primary fibroblasts were cultured from punch biopsy of healthy adult human skin, as described previously [17]. Briefly, human skin punch biopsies from sun-protected buttock (4 mm in diameter) were minced into small pieces and then digested by collagenase (5 mg/ml in αMEM) overnight at 37 °C. Tissue debris from digested skin biopsies was removed by 100-μm filters. Cells were cultivated in DMEM at 37 °C and 5% CO2 supplemented with 10% Fetal Bovine Serum (Sigma, MO). Cells were passaged no more than nine times. Cells were plated at 70–80% confluence and used one day later. All skin samples were obtained under a protocol approved by the University of Michigan Institutional Review Board. All volunteers provided written informed consent. All methods were performed in accordance with the relevant guidelines and regulations.

### RNA isolation and quantitative real-time RT-PCR

Total RNA from human skin primary fibroblasts was extracted using TRIzol reagent (Invitrogen, Carlsbad, CA). Reverse transcription of total RNA (100 ng) was carried out using a SYBR Green transcription kit (Applied Biosystems, Foster City, CA). Real-time PCR was performed on a 7300 Sequence Detector (Applied Biosystems,) using TaqMan Universal PCR Master Mix Reagents (Applied Biosystems,). All real-time PCR primers were purchased from RealTimePrimers.com (Elkins Park, PA). Target gene mRNA expression levels were normalized to the housekeeping gene 36B4 (acidic ribosomal phosphoprotein P0) [18] as an internal control for quantification.

### Transient transfection and luciferase assays

Human primary skin fibroblasts were transiently transfected by electroporation (Amaxa, Koeln, Germany). Transient transfection of Emerald Green Fluorescent Protein (EmGFP, The Vivid Colors™ ThermoFisher Waltham, MA) indicated that the transfection efficiency can be up to 80% in human primary skin fibroblasts by electroporation. siRNAs for the first set of YAP (GACAUCUUCUGGUCAGAGA) and TAZ (AGGUACUUCCUCAAUCACA) si RNAs and second set of YAP (CUGGUCAGAGAUACUUCUU) and TAZ (ACGUUGACUUAGGAACUUU) were purchased from Sigma Chemical Company (St. Louis, MO). The first set (AGCUCAAUGAGCAUGUUUAGACUTT) and the second set (ACAGCUCAAUUCGGACAACAAGAGT) Smad7 siRNAs were purchased from Origene (Rockville, MD). Smad3 siRNA (AATTCCTCGAGATAGGCCGTT) was purchased from Qiagen-Xeragen (Germantown, MD), and the specificity Smad3 antibody was confirmed by Smad3 siRNA (Additional file 1: Figure S7). Control siRNA (AATTGTCCGAACGTGTCACGT) were purchased from Qiagen (Chatsworth, CA). After transfection (48 h), total RNA and cellular protein were extracted, and mRNA and protein levels were determined by real-time RT-PCR and Western analysis, respectively, as described above. AP-1 reporter construct (pAP1-TA-Luc) was purchased from BD Biosciences Clontech (Palo Alto, CA). Smad binding element (SBE) luciferase reporter containing four repetitions of the GTCTAGAC Smad3/4 binding motif (SBEX4) was obtained from Bert Vogelstein of the Johns Hopkins Oncology Center, Baltimore, MD. Smad7 promoter/luciferase construct (− 613 to + 112) or deletion constructs in the binding sites of Smad3 and AP-1 were generously provided Dr. Rainer Heuchel [19]. Cells were co-transfected with a β-galactosidase expression vector, provide an internal standard for transfection efficiency. Aliquots containing identical β-galactosidase activity were used for each luciferase assay. Luciferase activity was measured using an enhanced luciferase assay kit (PharMingen International, San Diego, CA) according to the manufacturer’s protocol.

### Western analysis

Human skin primary fibroblasts were scraped from the culture dishes in WCE buffer (25 mM HEPES (pH 7.2), 75 mM NaCl, 2.5 mM MgCl_2_, 0.2 mM EDTA, 0.1% Triton X-100, 0.5 mM DTT, 20 mM β-glycerophosphate, 0.1 mM Na3VO4, 2 μg/ml leupeptin, and 100 μg/ml PMSF). The cells were frozen/thawed/vortexed three times, and whole-cell extracts were collected by centrifugation at 14,000×*g* for 10 min. Equal amounts of protein from whole cell extracts were resolved on 10% SDS-polyacrylamide gel (Invitrogen, CA) and transferred to PVDF membrane (Millipore, Bedford, MA) overnight. Next day, the membrane was blocked with PBST (0.1% Tween 20 in PBS) containing 5% milk for one hour. Primary antibodies were diluted in the PBST solution (1:200) was incubated with PVDF membrane for one hour at room temperature. Blots were washed three times with PBST solution and incubated with appropriate secondary antibody for one hour at room temperature. After washing three times with PBST, the blots were developed with ECF (Vistra ECF Western Blotting System, Amersham Pharmacia Biotech, Piscataway, NJ) following the manufacturer’s protocol. The intensities of each band were scanned by STORM PhosphorImager (Molecular Dynamics, Sunnyvale, CA). The intensities of each band were quantified using ImageQuant (GE HealthCare, Piscataway, NJ) and normalized using β-actin as a loading control.

### ProteinSimple capillary electrophoresis immunoassay

In some experiments, due to the limit of sample volumes, the protein levels were determined by ProteinSimple capillary electrophoresis immunoassay. ProteinSimple capillary electrophoresis immunoassay overcomes many of the technical drawbacks of Western analysis, while providing much greater sensitivity, in the low nanogram range. ProteinSimple capillary electrophoresis immunoassay was performed according to the ProteinSimple manufacture’s user manual. In brief, whole cell extract samples (800 ng/lane) were mixed with the kit provided master mix. The mixture was then heated at 95 °C for 5 min. The samples including primary antibodies, blocking reagent, HRP-conjugated secondary antibodies, chemiluminescent substrate, and separation and stacking matrices were also dispensed to designated wells plate according to the ProteinSimple manufacture’s user manual. The electrophoresis, blocking, washing, and immunodetection steps took place in the capillary system (ProteinSimple Wes, ProteinSimple, Santa Clare, CA) and were fully automated with instrument default settings. Corresponding protein bands from digital images were identified based on the molecular weight. The intensities of each protein were analyzed by quantification with Compass software (ProteinSimple) after normalization by β-actin (loading control).

### Immunohistology and phalloidin staining

Immunohistology was performed as described previously [12]. Briefly, fibroblasts were fixed in 2% paraformaldehyde for one hour at room temperature and were incubated with 0.5% Nonidet P-40 and then blocked with 2% bovine serum albumin (BSA). The cells were washed with PBS three times and incubated with primary antibodies (YAP/TAZ and Smad7, Santa Cruz Biotechnology, Santa Cruz, CA; Phospho Smad3, Abcam, Cambridge, MA) for 1 h at room temperature, followed by incubation with Super Sensitive MultiLink (BioGenex, Fremont CA) for 10 min and streptavidin-conjugated AlexaFluor 594 or 488 (Invitrogen-Molecular Probes, San Diego, CA) for 10 min. Mounting medium with DAPI was added to stain cell nuclei. Corresponding IgG isotype (negative control) show no specific staining (data not shown). For phalloidin (Sigma, St. Louis, MO, USA) staining, fibroblasts were washed with PBS and were fixed in 2% paraformaldehyde for 30 min followed by phalloidin staining for one hour.

### Electrophoretic mobility shift assay (EMSA)

EMSA were performed as described previously [20] using nuclear extracts from human skin primary fibroblasts. Oligonucleotides for EMSA probe, designed from the Smad7 promoter, were as follows: Smad binding element (SBE) probe, 5’AAGCGACAGGGTGTCTAGACGG3’; AP-1 probe, 5’CACGTGACGAGGCCGGAGCCGG3’. AP-1 consensus (5’CGCTTGATGACTCAGCCGGAA3’) and mutant (5’CGCTTGATGACTTGGCCGGAA3’) oligonucleotides were purchased from Santa Cruz Biotechnology (Santa Cruz, CA). For competition experiments, a 50-fold molar excess of cold competitors (AP-1 or SBE) were pre-incubated with nuclear extract for 30 min on ice before labeled probes were added. The gel was transferred to 3MM Whatman paper, vacuum-dried and scanned by STORM PhosphorImager (Molecular Dynamics, Sunnyvale, CA).

### Statistical analysis

Comparisons among treatment groups were made with the paired t-test (two groups) or the repeated measures of ANOVA (more than two groups). Multiple pair-wise comparisons were made with the Tukey Studentized Range test. All *p* values are two-tailed and considered significant when <0.05.

## Results

### YAP/TAZ knockdown impairs TGF-β1-induced Smad3 phosphorylation and Smad reporter activity

In the course of investigating the impact of YAP/TAZ knockdown on the regulation of cell proliferation in primary human dermal fibroblasts [21], we unexpectedly observed substantial inhibition of TGF-β1-induced Smad3 phosphorylation (Fig. 1a and b). We observed that knockdown of YAP or TAZ alone did not have any notable effect on TGF-β1-induced Smad3 phosphorylation. In contrast, combined knockdown of YAP and TAZ significantly impaired TGF-β1-induced Smad3 phosphorylation. This observation likely reflects compensatory actions of YAP and TAZ. Consistent with this possibility, we found that the levels of YAP and TAZ mRNA were similar, in primary human dermal fibroblasts (Fig. 1c). Additionally, to confirm the specificity of the effects of siRNA-mediated knockdown of YAP and TAZ, we utilized a different set of YAP and TAZ siRNAs (YAP/TAZ siRNAs#2) (Fig. 1d) and found similar impairment of TGF-β1-induced Smad3 phosphorylation (Fig. 1e). In agreement with these data, YAP/TAZ knockdown inhibited TGF-β1-induced Smad3 activity, measured by Smad binding element (SBE) luciferase reporter assay (Fig. 1f). To further investigate the mechanism by which YAP/TAZ knockdown inhibits activation of Smad3, we inhibited YAP/TAZ nuclear localization by treatment of fibroblasts with latrunculin-A, which causes disassembly of the actin cytoskeleton (Fig. 1g) [22]. Inhibition of YAP/TAZ nuclear localization resulted in significant reduction of TGF-β1-induced Smad3 phosphorylation (Fig. 1h, top right panel), indicating that nuclear YAP/TAZ is involved in regulation of TGF-β/Smad3 signaling. Together, these data demonstrate that intact TGF-β/Smad3 signaling requires both YAP and TAZ in primary human dermal fibroblasts.

### YAP/TAZ knockdown inhibits TGF-β target gene expression

**Fig. 2:**
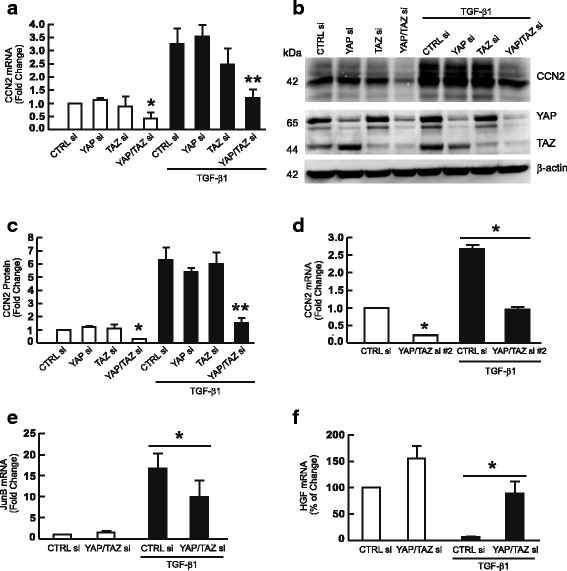
YAP/TAZ knockdown inhibits expression of TGF-β target genes CCN2, JunB, and HGF. Primary adult human dermal fibroblasts were transfected with non-specific control siRNA or YAP and/or TAZ siRNAs (400 nM). 32 h after transfection, cells were treated with vehicle (PBS) or TGF-β1 (5 ng/ml) for 16 h. Whole cell extracts were prepared 48 h after transfection. **a** CCN2 mRNA levels. *N* = 4, *p < 0.05 vs CTRL siRNA, **p < 0.05 vs CTRL siRNA with TGF-β1. **b** and **c** CCN2 protein levels. *N* = 3 *p < 0.05 vs CTRL siRNA, **p < 0.05 vs CTRL siRNA with TGF-β1. Full-length blots are presented in Additional file 1: Figure S3. **d** CCN2 mRNA levels are reduced by YAP/ TAZ siRNA #2. *N* = 3, *p < 0.05 vs CTRL siRNA, **p < 0.05 vs CTRL siRNA with TGF-β1. **e** JunB mRNA levels. *N* = 3, *p < 0.05 vs CTRL siRNA with TGF-β1. **f** HGF mRNA levels. *N* = 3, *p < 0.05 vs CTRL siRNA with TGF-β1. mRNA levels were quantified by real-time RT-PCR and normalized to mRNA for housekeeping gene 36B4. Protein levels were determined by Western blots. Protein levels were normalized by β-actin as a loading control. All data are mean±SEM

To further explore the role of YAP/TAZ in TGF-β/Smad3 signaling, we investigated the effects of YAP and/or TAZ knockdown on TGF-β target gene expression. In human skin dermal fibroblasts, TGF-β is a potent stimulator of CCN2 (also known as connective tissue growth factor, CTGF) expression [12] We found that combined knockdown of YAP and TAZ, but not separate knockdown of YAP or TAZ, significantly reduced TGF-β1-induced CCN2 mRNA (Fig. 2a) and protein (Fig. 2b and c) levels. Similar results were obtained with a different set of TAZ siRNAs (YAP/TAZ siRNAs #2) (Fig. 2d). We also found that combined YAP and TAZ knockdown reduced CCN2 levels in the absence of added TGF-β (Fig. 2a-d), suggesting direct regulation of CCN2, as previously reported [23, 24]. Therefore, we investigated two other TGF-β target genes, JunB [25–27] and hepatocyte growth factor (HGF) [28–30]. Combined knockdown of YAP and TAZ significantly reduced TGF-β1 induction of both JunB (Fig. 2e) and HGF (Fig. 2f). These data further demonstrate that knockdown of YAP/TAZ impairs TGF-β signaling, and thus inhibits TGF-β target gene expression.

### Knockdown of YAP/TAZ induces Smad7, an inhibitor of TGF-β/Smad signaling

**Fig. 3:**
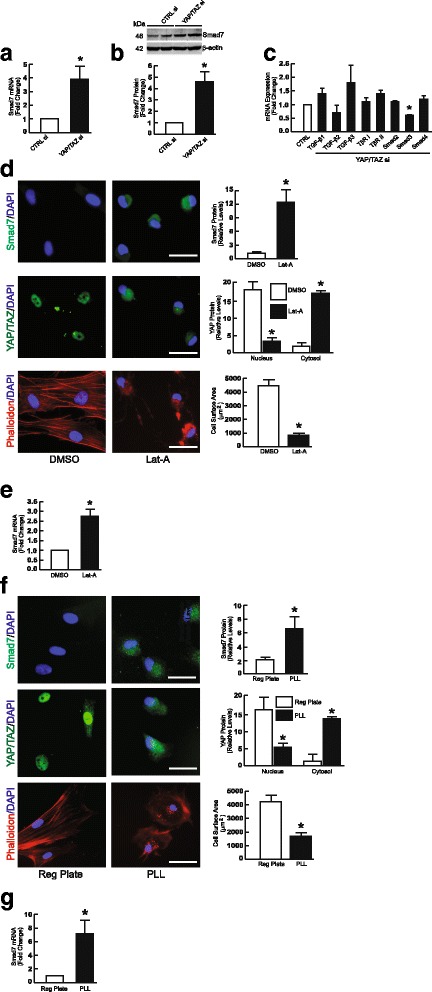
Knockdown of YAP/TAZ or inhibition of YAP/TAZ nuclear localization induces inhibitory Smad7. Primary adult dermal fibroblasts were transfected with non-specific control or YAP/TAZ siRNAs (400 nM). Whole cell extracts were prepared 48 h after transfection. **a** Smad7 mRNA levels. mRNA levels were quantified by real-time RT-PCR. mRNA levels were normalized to mRNA for housekeeping gene 36B4. *N* = 5, *p < 0.05 vs CTRL siRNA. **b** Smad7 protein levels. Protein levels were determined by Western blot and normalized by β-actin (loading control). *N* = 3, *p < 0.05 vs CTRL siRNA. Full-length blots are presented in Additional file 1: Figure S4. **c** TGF-β pathway components mRNA expression. *N* = 3–6. **d** Actin cytoskeleton disassembly induces Smad7 protein. Primary adult human dermal fibroblasts were treated with vehicle (DMSO) or latrunculin A (Lat = A, 30 nM) for 24 h, and then cells were immunostained for Smad7 (top panel, green), YAP/TAZ (middle panel, green), and phalloidin (lower panel, red). Nuclei were stained with DAPI (blue). Scale bars = 25 μm. The staining intensities and cell surface areas were quantified by ImageJ software. *p < 0.05 vs DMSO. All images are representative of three independent experiments. **e** Actin cytoskeleton disassembly induces Smad7 mRNA. mRNA levels were quantified by real-time RT-PCR and normalized to mRNA for housekeeping gene 36B4. *N* = 3, *p < 0.05 vs DMSO. **f** Limiting cell spreading induces Smad7 protein. Fibroblasts were plated on standard tissue culture plates (left panels) or poly-L-lysine (PLL) coated plates (right panels). 24 h later, cells were immunostained for Smad7 (top panel, green), YAP/TAZ (middle panel, green), and phalloidin (low panel, red). Nuclei were stained with DAPI (blue). Scale bars = 25 μm. The staining intensities and cell surface areas were quantified by ImageJ software. *p < 0.05 vs standard tissue culture plate. All images are representative of three independent experiments. **g** Limiting cell spreading induces Smad7 mRNA. mRNA levels were quantified by real-time RT-PCR and normalized to mRNA for housekeeping gene 36B4. *N* = 3, *p < 0.05 vs DMSO. All data are mean±SEM

**Fig. 4:**
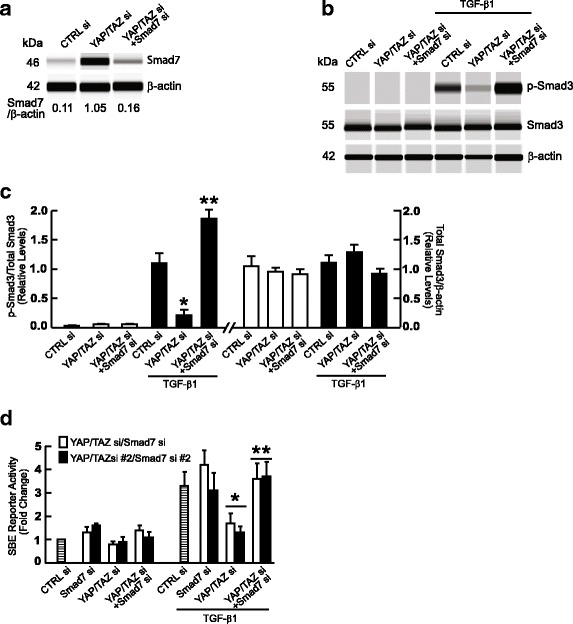
Knockdown of Smad7 reverses inhibition of TGF-β/Smad3 signaling by YPA/TAZ silencing. **a** Reduction of elevated Smad7 by siRNA-mediated knockdown. Smad7 protein levels were determined by ProteinSimple capillary electrophoresis immunoassay. Data are representative of two independent experiments. **b** and **c** Restoration of TGF-β1-induced Smad3 phosphorylation by Smad7 knockdown. Fibroblasts were transfected with the indicated siRNAs and 48 h treated with TGF-β1 (5 ng/ml) for 1 h. Protein levels of total Smad3, phospho-Smad3, and beta-actin (loading control) were determined by ProteinSimple capillary electrophoresis immunoassay. *N* = 2, *p < 0.05 vs CTRL siRNA with TGF-β1, **p < 0.05 vs YAP/TAZ siRNA with TGF-β1. **d** Restoration of TGF-β/Smad3 activity by Smad7 knockdown. Fibroblasts were co-transfected with non-specific control or YAP/TAZ siRNAs (400 nM) and/or Smad7 siRNAs along with Smad Binding Element (SBE) luciferase reporter and β-galactosidase expression vector. 32 h later, cells were treated with vehicle (PBS) or TGF-β1 (5 ng/ml) for 16 h. SBE luciferase activities were determined 48 h after transfection and normalized to β-galactosidase activity. Bars indicate fold change in SBE reporter activity relative to control siRNA. *N* = 3, *p < 0.05 vs control siRNA with TGF-β1,**p < 0.05 vs YAP/TAZ siRNA with TGF-β1. All data are expressed as mean±SEM

To explore the potential mechanism by which knockdown of YAP/TAZ impairs TGF-β/Smad signaling, we determined the effect of YAP/TAZ knockdown on expression of the primary TGF-β pathway components, such as TGF-β ligands, TGF-β receptors, and intracellular Smads. Interestingly, we found that YAP/TAZ knockdown resulted in significant induction of Smad7, a potent inhibitor of TGF-β signaling. Both Smad7 mRNA (Fig. 3a) and protein (Fig. 3b) levels were induced. We also observed that YAP/TAZ knockdown reduced Smad3 mRNA expression (Fig. 3c), however, Smad3 protein levels were not altered (see Fig. 4b). No other changes in TGF-β pathway components were observed (Fig. 3c). We also observed that inhibition of YAP/TAZ nuclear localization induced Smad7 mRNA and protein. Nuclear localization was inhibited by either Lat-A (Fig. 3d and e), as described above (Fig. 1g and h), or by culture of fibroblasts on poly-L-lysine (PLL) coated dishes (Fig. 3f and g), which allows integrin-independent attachment and limits cell spreading [31]. In either condition, inhibition of YAP/TAZ nuclear translocation resulted in significant induction of Smad7 protein and mRNA. Together, these data demonstrate that knockdown of YAP/TAZ or inhibition of YAP/TAZ nuclear translocation induces inhibitory Smad7.

### Induction of Smad7 by YAP/TAZ siRNA mediates impaired TGF-β/Smad signaling

We next investigated whether induction of Smad7 is responsible for inhibition of TGF-β signaling, which occurs in response to knockdown of YAP/TAZ. We first confirmed that Smad7 siRNA was able to efficiently inhibit induction of Smad7 by YAP/TAZ knockdown (Fig. 4a). Importantly, we found that knockdown of Smad7 was sufficient to prevent inhibition of TGF-β1-induced Smad3 phosphorylation in fibroblasts that have knockdown of YAP/TAZ (Fig. 4b and c). In addition, prevention of Smad7 elevation significantly restored SBE luciferase reporter activity in in fibroblasts with YAP/TAZ knockdown (Fig. 4d). Together, these data demonstrate that knockdown of YAP/TAZ or inhibition of YAP/TAZ nuclear translocation induces inhibitory Smad7, which in turn impairs TGF-β/Smad signaling.

### Knockdown of YAP/TAZ induces AP-1, the major driving force for Smad7

**Fig. 5:**
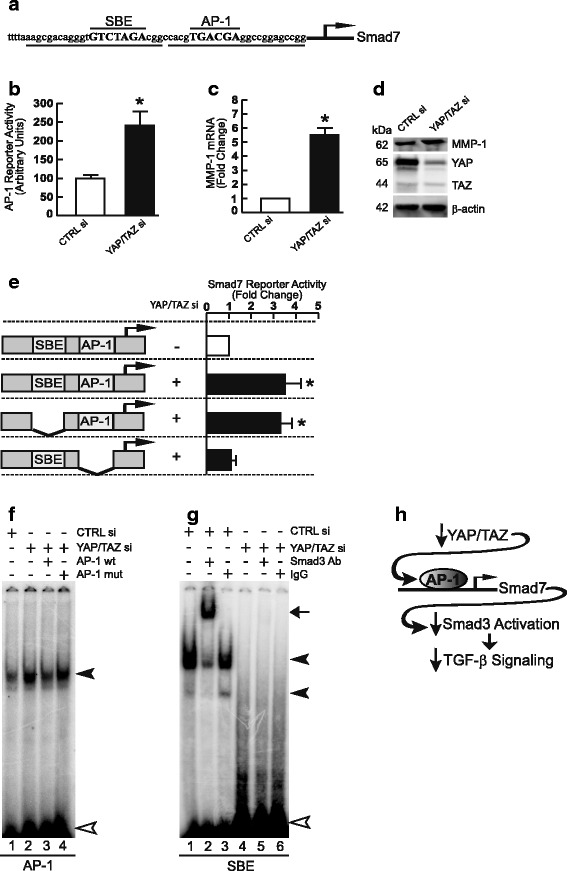
Induction of Smad7 by knockdown of YAP/TAZ is mediated by AP-1. **a** Nucleotide sequence of the Smad7 proximal promoter. Smad Binding Element (SBE) and Activator Protein 1 (AP-1) binding sites are marked in capital letters. Heavy lines under the sequence denote EMSA probes containing SBE and AP-1 binding elements. Arrow indicates transcription start site. **b** Primary adult human dermal fibroblasts were co-transfected with non-specific control or YAP/TAZ siRNAs (400 nM), AP-1 reporter construct, and β-galactosidase expression vector. AP-1 reporter activities were determined 48 h after transfection and normalized to β-galactosidase activity. *N* = 3, *p < 0.05 vs control siRNA. **c** and **d** Fibroblasts were co-transfected with non-specific control or YAP/TAZ siRNAs (400 nM). Whole cell extracts were prepared 48 h after transfection. **c** MMP-1 mRNA levels and (**d**) MMP-1 protein levels were analyzed by real-time RT-PCR and Western blot, respectively. mRNA levels were normalized to mRNA for housekeeping gene 36B4. Full-length Western blots are presented in Additional file 1: Figure S5A. *p < 0.05 vs control siRNA. **e** Fibroblasts were co-transfected with non-specific control or YAP/TAZ siRNAs (400 nM), wild-type (− 613 to + 112) or mutant Smad7 promoter/luciferase reporter constructs as depicted in the figure, and β-galactosidase expression vector. Smad7 promoter luciferase activities were determined 48 h after transfection and normalized to β-galactosidase activity. Bars indicate fold change of Smad7 promoter activity relative to control siRNA. *N* = 3–5, *p < 0.05 vs control siRNA. **f** and **g** Fibroblasts were transfected with non-specific control or YAP/ TAZ siRNAs (400 nM) for 48 h. Nuclear extracts were prepared, and DNA/protein complex formation was analyzed by EMSA using AP-1 (**f**) and SBE (**g**) probes, as indicated in Fig. 5a (underlined). Closed triangles indicate specific retarded complexes. Open triangle indicates non-specific bands. Arrow indicates Smad3 super-shift band. Results are representative of three experiments. Full-length images are presented in Additional file 1: Figure S5B and S5C. All data are expressed as mean±SEM. **h** Schematic of cross-talk between YAP/TAZ and TGF-β/Smad pathways involving AP-1 regulation of Smad7

Finally, we explored the mechanism by which knockdown of YAP/TAZ induces Smad7. The human Smad7 gene promoter contains binding sites for Smad3 and activator protein-1 (AP-1) (Fig. 5a) [19, 32]. In human dermal fibroblasts, these two transcription factors act independently to regulate Smad7 expression. Smad3 stimulates Smad7 expression in response to TGB-β, while AP-1 stimulates Smad7 in response to exposure to ultraviolet irradiation [31]. Since knockdown of YAP/TAZ impairs Smad3 activity, we investigated whether knockdown of YAP/TAZ leads to increased AP-1 activity. Indeed, knockdown of YAP/TAZ significantly elevated AP-1 reporter activity (Fig. 5b). Knockdown of YAP/TAZ also increased expression of matrix metalloproteinase-1 (MMP-1) (Fig. 5c and d), which is well-characterized AP-1 target gene. To examine the role AP-1 in Smad7 induction, Smad7 proximal promoter luciferase reporter constructs, containing the AP-1 and the Smad3 binding sites, were transiently transfected into fibroblasts with and without YAP/TAZ knockdown. Knockdown of YAP/TAZ resulted in significant activation (3.4-fold) of Smad7 promoter activity, and this activation was nearly abolished by deletion of the AP-1 binding site, while deletion of the Smad3 binding site had no effect (Fig. 5e).

We next investigated whether knockdown of YAP/TAZ increases the binding to the AP-1 element in the Smad7 gene promoter, using electrophoretic mobility shift assays (EMSA). The double-stranded DNA probe containing the AP-1 binding sequence in the Smad7 promoter is shown in Fig. 5a (underlined). As shown in Fig. 5f, knockdown of YAP/TAZ resulted in substantial increase binding to the AP-1/Smad7 probe (lane 2). This complex was substantially reduced by excess unlabeled consensus AP-1 probe (Fig. 5f, lane 3), but not by the mutant AP-1 probe (Fig. 5f, lane 4), indicating that binding was specific. As expected, knockdown of YAP/TAZ significantly decreased Smad 3 binding to the SBE probe of the Smad7 promoter (Fig. 5g). This finding is consistent lack of effect of removal of the SBE in the Smad7 promoter (Fig. 5e) and impairment of TGB-β/Smad3 signaling (Figs. 1, 2 and 3) by knockdown of YAP/TAZ. Together these data indicate that knockdown of YAP/TAZ induces AP-1, which in turn mediates induction of Smad7.

## Discussion

Several recent studies have demonstrated cross-talk between YAP/TAZ and the TGF-β pathway. For example, nuclear YAP and TAZ have been found to physically and functionally interact with Smad transcriptional complexes to modulate their activity [33, 34] and stability, through ubiquitin-mediated degradation [35, 36]. In addition, YAP/TAZ have also been reported to interact with Smad7, and thereby potentiate its inhibitory activity [35, 36]. Thus, YAP/TAZ can modulate TGF-β/Smad pathway through interaction with Smads complexes. Our data reveal a distinct mechanism of cross-talk between YAP/TAZ and TGF-β/Smad signaling that involves transcriptional regulation of Smad7 expression. We found that knockdown of YAP/TAZ or inhibition of YAP/TAZ nuclear localization reduces Smad3 phosphorylation and activity. This inhibition results from up-regulation of Smad7 via an AP-1-dependent mechanism.

TGFβ family members regulate diverse physiological and pathological events through Smad proteins, the central modulators of TGF-β signaling. Although Smad7 induction by TGF-β itself is well characterized, the mechanisms underlying Smad7 regulation by other pathways are incompletely understood. Our data indicate that YAP/TAZ functions as an endogenous repressor of Smad7 gene expression, in human dermal fibroblasts. This conclusion is consistent with the relative levels of YAP/TAZ and Smad7 observed in both human dermis (Additional file 1: Figure S1A) and human primary human dermal fibroblasts (Additional file 1: Figure S1B).

We found that combined knockdown of YAP and TAZ, but not separate knockdown, induced Smad7 and impairs TGF-β signaling. Also, separate knockdown of YAP or TAZ did not result in induction of the other. These findings likely reflect compensatory effects of YAP and TAZ on Smad7 expression. YAP and TAZ have highly homologous structures and redundancy of functions is not unexpected. A recent ChIP-seq analysis of genome-wide promoter and enhancer regions found that the binding sites of YAP and TAZ largely overlap with each other [37].

Our findings raise an interesting question regarding the mechanism by which reduction of YAP/TAZ leads to up-regulation of AP-1 activity. YAP/TAZ knockdown did not significantly alter expression levels of AP-1 family (JUN and FOS proteins) members (Additional file 1: Figure S6). The AP-1 family member JunB is regulated by TGF-β/Smad3 and silencing of YAP/TAZ reduced TGF-β-induced JunB expression (Fig. 2e). These data indicate that JunB is not required for up-regulation of AP-1 activity, which results from knockdown of YAP/TAZ. This conclusion is consistent with our previous findings that c-Jun and c-Fos are the principal components of the AP-1 complex in human skin [38–40]. In human skin primary fibroblasts, many AP-1 target genes, such as multiple MMPs, are largely regulated by c-Jun [41, 42]. In addition to transcription, AP-1 activity is regulated by dimer composition, post-translational modifications, and interactions with other proteins [43]. Which of these mechanisms are involved in regulation of AP-1 activity by YAP/TAZ remains to be determined.

Interestingly, a recent study has reported that YAP expression is regulated by c-Jun [44]. Transient transfection of c-Jun significantly increased YAP mRNA levels. Conversely, YAP mRNA levels were markedly reduced in c-Jun deficient cells. Retroviral-mediated re-introduction of c-Jun restored YAP mRNA expression levels. Moreover, a recent study demonstrated that YAP/TAZ/TEAD and AP-1 form a complex in the nucleus that synergistically activates target genes involved in oncogenic growth [37]. Thus, emerging data demonstrate that AP-1 is involved in both YAP/TAZ expression and function. Our data extend these observations by revealing that lower levels of YAP/TAZ enhance AP-1 activity. Given the functional cooperativity between YAP/TAZ and AP-1, one may speculate that reduction of YAP/TAZ leads to a compensatory increase of AP-1 activity, in dermal fibroblasts.

## Conclusions

YAP/TAZ knockdown or inhibition of YAP/TAZ nuclear localization induces Smad7 via activation of AP-1, which in turn impairs TGF-β/Smad signaling (Fig. 5h). These data reveal a new mechanism for cross-talk between YAP/TAZ TGF-β signaling pathways.

## Additional file

Additional file 1: **Figure S1.** Smad7, YAP, and TAZ mRNA expression in human skin dermis in vivo and primary dermal fibroblasts in vitro. (A) Human skin dermis was prepared by cutting off epidermis at a depth of 1 mm by cryostat. Dermal total RNA was prepared using a commerical kit (RNeasy midikit, Qiagen, Chatsworth, CA). *N* = 8. (B) Total RNA from primary dermal dibroblasts was extracted using TRIzol reagent (Invitrogen, Carlsbad, CA). *N* = 3 mRNA levels for Smad7, YAP, and TAZ were determined by real-time RT-PCR. mRNA levels were normalized to mRNA for 36B4, a ribosomal protein used as an internal control for quantitation. Data are expressed as mean + SEM, *p < 0.05 vs Smad7. **Figure S2.** Full-length Western blots for Fig. 1a. **Figure S3.** Full-length Western blots for Fig. 2b. **Figure S4.** Full-length Western blots for Fig. 3b. **Figure S5 A.** Full-length Western blots for Fig. 5d. **B.** Full-length Western blots for Fig. 5f. **C.** Full-length Western blots for Fig. 5g. **Figure S6** YAP/TAZ knockdown did not alter AP-1 family transcription factors mRNA expression. Primary dermal fibroblasts were transfected with non-specific control siRNA or YAP/TAZ siRNAs (400 nM) for 48 h. AP-1 family transcription factors mRNA levels were quantified by real-time RT-PCR. mRNA levels were normalized to mRNA for 36B4, a ribsomal protein used as an internal control for quantitation. *N* = 4, data are expressed as mean + SEM. **Figure S7.** Smad3 antibody specificity testing by Smad3. Primary dermal fibroblasts were transfected with with non-specific control siRNA or Smad3 siRNA (400 nM). 48 h after transfection, cells were treated with TGF-β1 (ng/ml) for one hour and whole cell extract was prepared. Protein levels of phospho-Smad3 were determined by Capillary electrophoresis immunoassay and normalized to β-actin (loading control). Band intensities were quantified by Compass software. Bands show representative digital images. (PDF 175 kb)

## Abbreviations

CCN2/CTGF: Connective tissue growth factor
ECM: Extracellular matrix
EMSA: Electrophoretic mobility shift assay
TAZ: Transcriptional coactivator with PDZ-binding motif
YAP: Yes-associated protein
