# Supplementary material for: YAP/TAZ regulates TGF-β/Smad3 signaling by induction of Smad7 via AP-1 in human skin dermal fibroblasts
Source: Cell Commun Signal. 2018 Apr 25;16:18. doi: 10.1186/s12964-018-0232-3 (PMC5918965; doi:10.1186/s12964-018-0232-3)
Supplement: Supplementary file 1 — Figure S1. Smad7, YAP, and TAZ mRNA expression in human skin dermis in vivo and primary dermal fibroblasts in vitro. (A) Human skin dermis was prepared by cutting off epidermis at a depth of 1 mm by cryostat. Dermal total RNA was prepared using a commerical kit (RNeasy midikit, Qiagen, Chatsworth, CA). N = 8. (B) Total RNA from primary dermal dibroblasts was extracted using TRIzol reagent (Invitrogen, Carlsbad, CA). N = 3 mRNA levels for Smad7, YAP, and TAZ were determined by real-time RT-PCR. mRNA levels were normalized to mRNA for 36B4, a ribosomal protein used as an internal control for quantitation. Data are expressed as mean + SEM, *p < 0.05 vs Smad7. Figure S2. Full-length Western blots for Fig. 1a. Figure S3. Full-length Western blots for Fig. 2b. Figure S4. Full-length Western blots for Fig. 3b. Figure S5 A. Full-length Western blots for Fig. 5d. B. Full-length Western blots for Fig. 5f. C. Full-length Western blots for Fig. 5g. Figure S6 YAP/TAZ knockdown did not alter AP-1 family transcription factors mRNA expression. Primary dermal fibroblasts were transfected with non-specific control siRNA or YAP/TAZ siRNAs (400 nM) for 48 h. AP-1 family transcription factors mRNA levels were quantified by real-time RT-PCR. mRNA levels were normalized to mRNA for 36B4, a ribsomal protein used as an internal control for quantitation. N = 4, data are expressed as mean + SEM. Figure S7. Smad3 antibody specificity testing by Smad3. Primary dermal fibroblasts were transfected with with non-specific control siRNA or Smad3 siRNA (400 nM). 48 h after transfection, cells were treated with TGF-β1 (ng/ml) for one hour and whole cell extract was prepared. Protein levels of phospho-Smad3 were determined by Capillary electrophoresis immunoassay and normalized to β-actin (loading control). Band intensities were quantified by Compass software. Bands show representative digital images. (PDF 175 kb) [file 12964_2018_232_MOESM1_ESM.pdf]

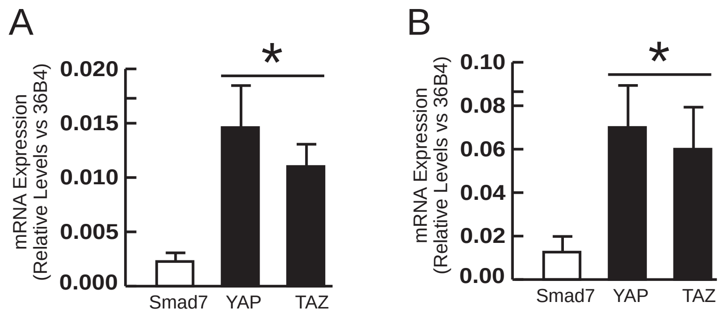

Figure S1

Smad7, YAP, and TAZ mRNA expression in human skin dermis *in vivo* and primary dermal fibroblasts *in vitro*. (A) Human skin dermis was prepared by cutting off epidermis at a depth of 1mm by cryostat. Dermal total RNA was prepared using a commerical kit (RNeasy midikit, Qiagen, Chatsworth, CA). N=8. (B) Total RNA from primary dermal dibroblasts was extracted using TRIzol reagent (Invitrogen, Carlsbad, CA). N=3 mRNA levels for Smad7, YAP, and TAZ were determined by real-time RT-PCR. mRNA levels were normalized to mRNA for 36B4, a ribosomal protein used as an internal control for quantitation. Data are expressed as mean$\pm$SEM, *$p<0.05$ vs Smad7.

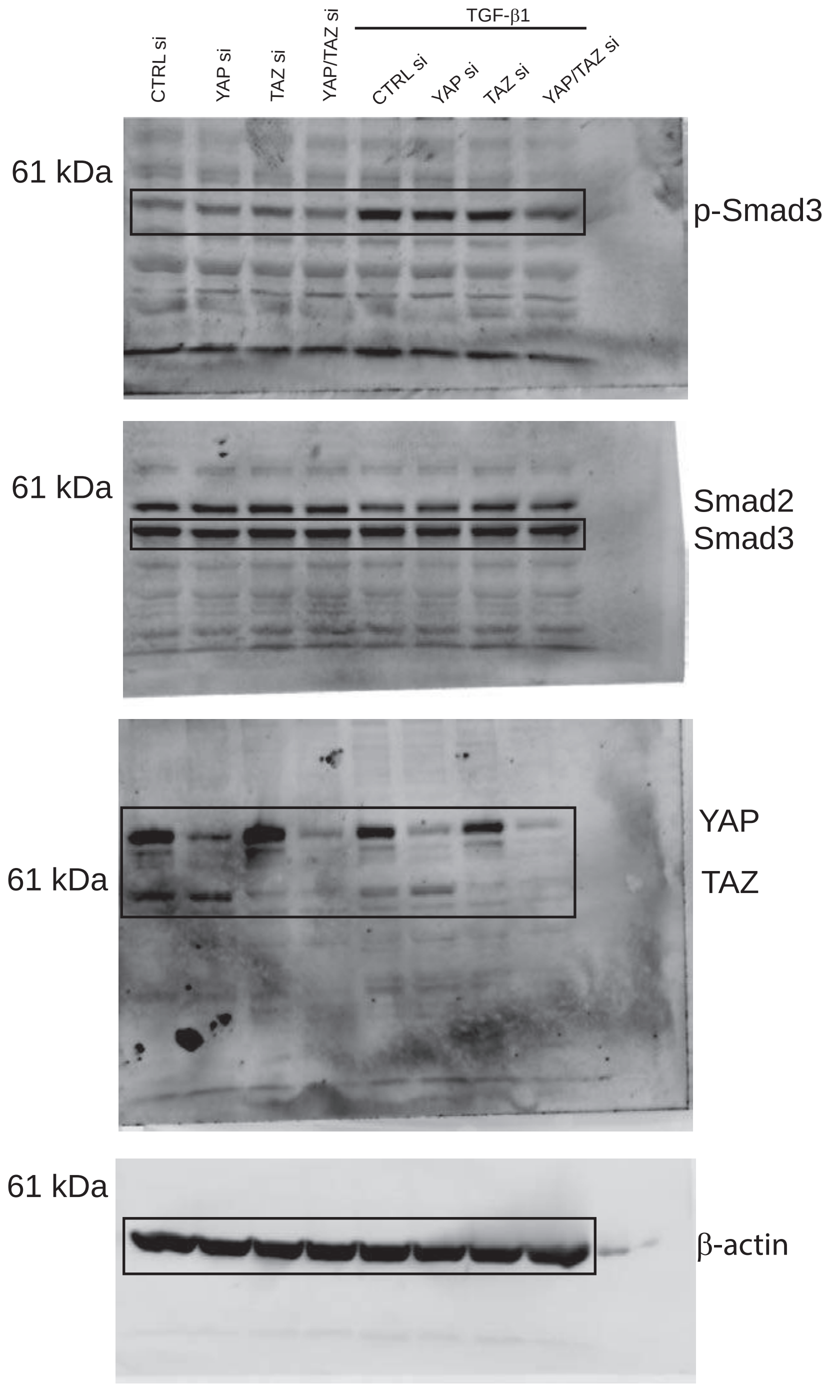

Figure S2. Full-length Western blots for Figure1A.

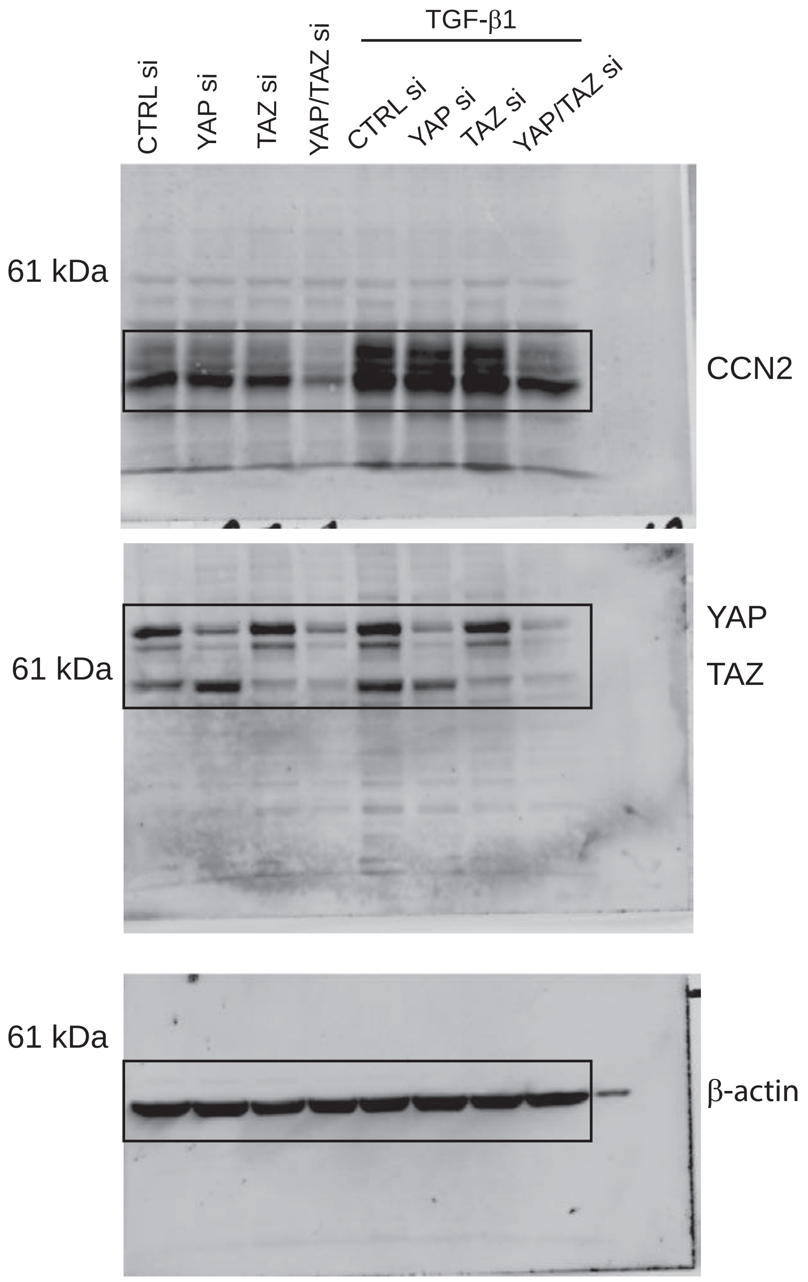

Figure S3. Full-length Western blots for Figure 2B.

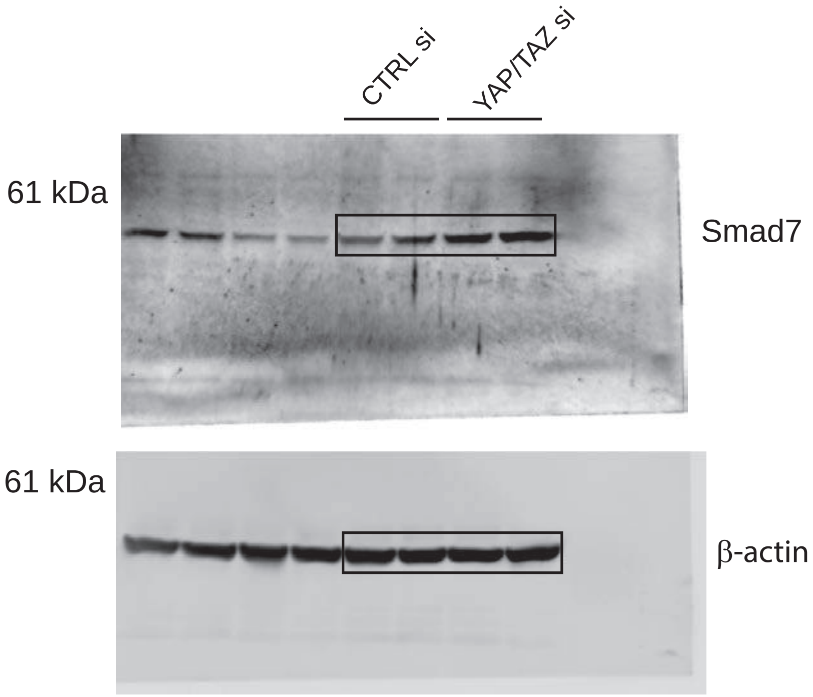

Figure S4. Full-length Western blots for Figure 3B.

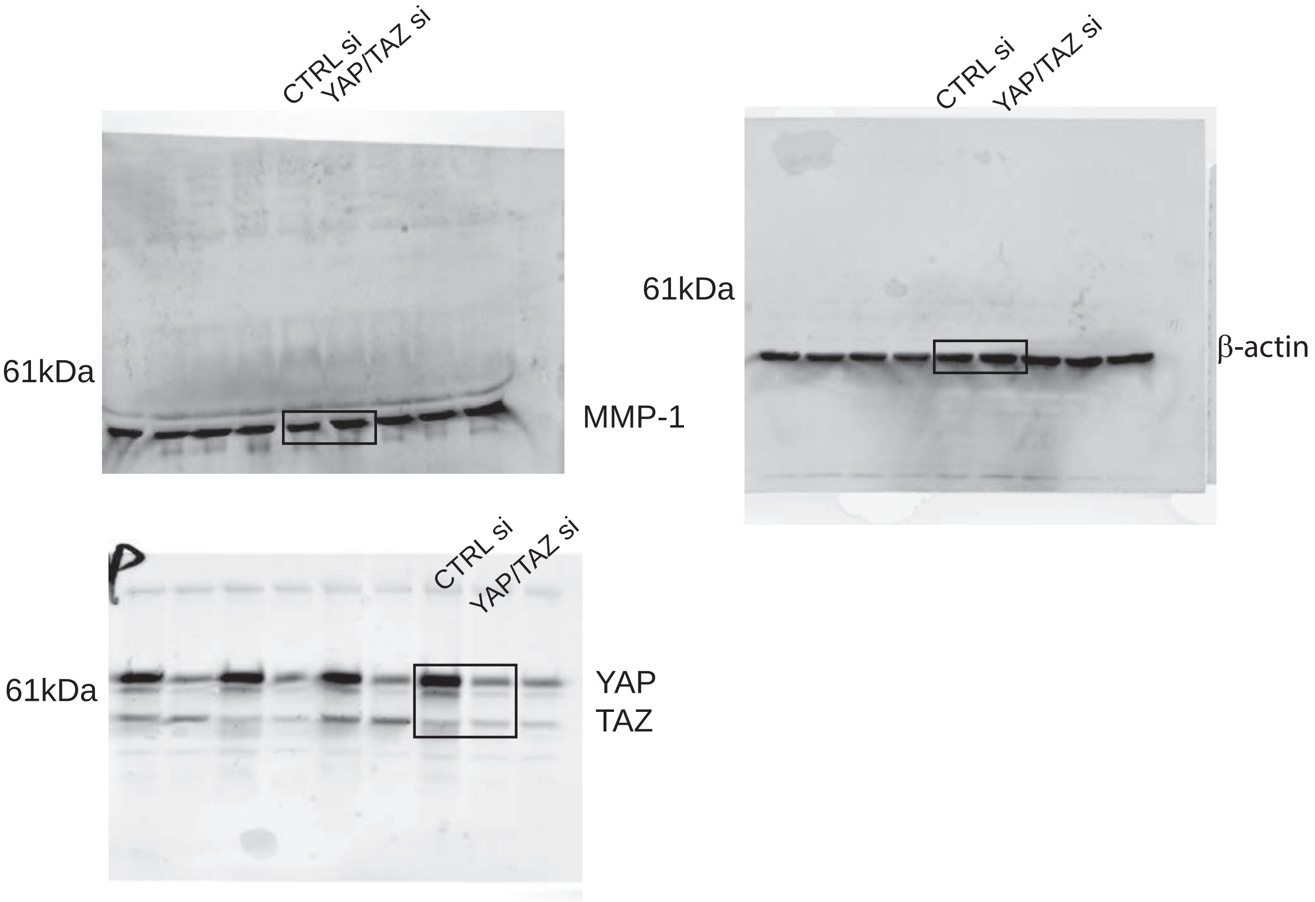

Figure S5A. Full-length Western blots for Figure 5D.

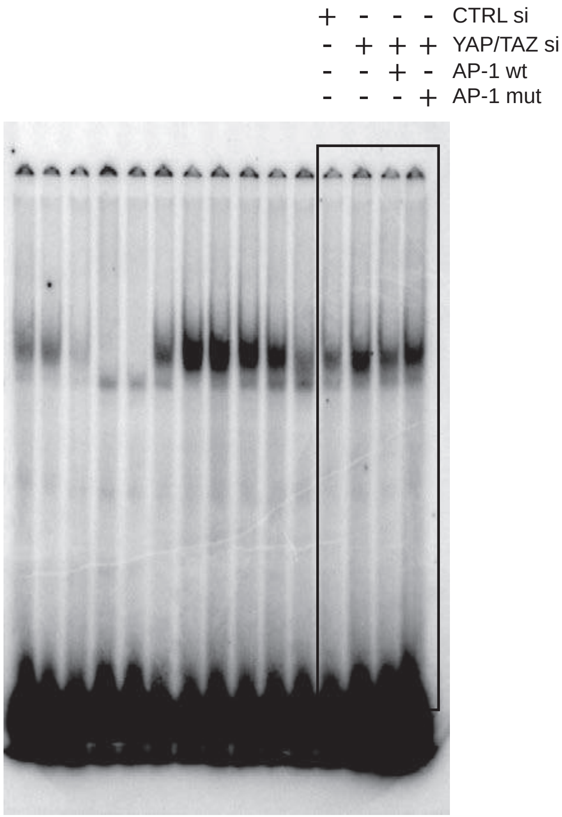

Figure S5B

Full-length Western blots for Figure 5F.

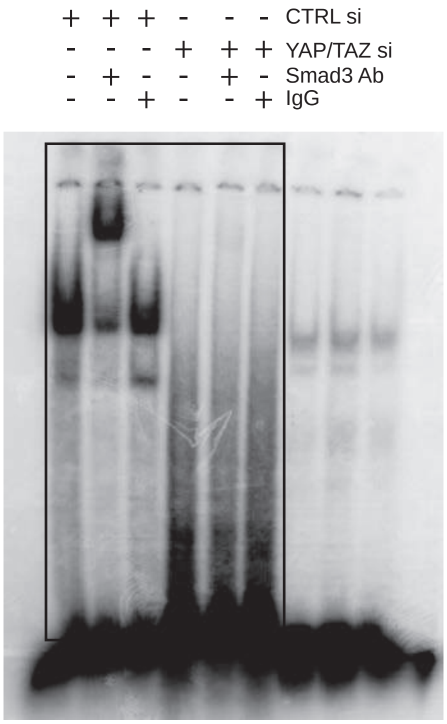

Figure S5C

Full-length Western blots for Figure 5G.

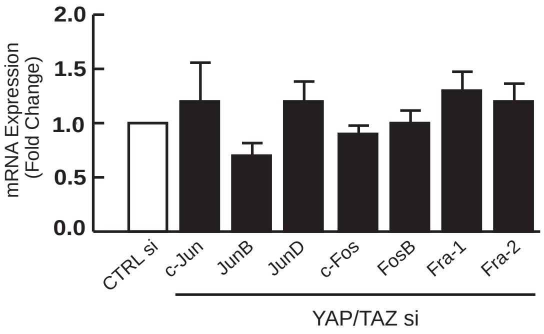

Figure S6

YAP/TAZ knockdown did not alter AP-1 family transcription factors mRNA expression. Primary dermal fibroblasts were transfected with non-specific control siRNA or YAP/TAZ siRNAs (400nM) for 48 hours. AP-1 family transcription factors mRNA levels were quantified by real-time RT-PCR. mRNA levels were normalized to mRNA for 36B4, a ribsomal protein used as an internal control for quantitation. N=4, data are expressed as mean$\pm$SEM.

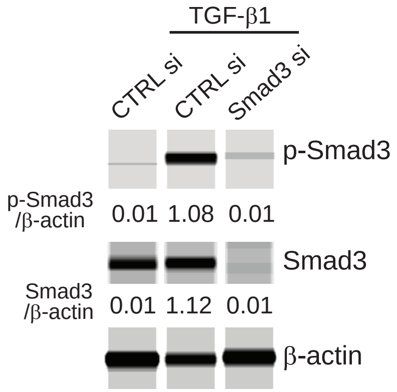

Figure S7

Smad3 antibody specificity testing by Smad3. Primary dermal fibroblasts were transfected with with non-specific control siRNA or Smad3 siRNA (400nM). 48 hours after transfection, cells were treated with TGF-β1 (ng/ml) for one hour and whole cell extract was prepared. Protein levels of phospho-Smad3 were determined by Capillary electrophoresis immunoassay and normalized to β-actin (loading control). Band intensities were quantified by Compass software. Bands show representative digital images.
